# Supplementary material for: Muscle abnormalities worsen after post-exertional malaise in long COVID
Source: Nat Commun. 2024 Jan 4;15:17. doi: 10.1038/s41467-023-44432-3 (PMC10766651; doi:10.1038/s41467-023-44432-3)
Supplement: Supplementary file 1 — Supplementary Data File [file 41467_2023_44432_MOESM1_ESM.pdf]

Supplementary information to:
Muscle Abnormalities Worsen After Post-Exertional Malaise in Long COVID

Brent Appelman[1,2]†, Braeden T. Charlton[3,4]†, Richie P. Goulding[3,4], Tom Kerkhoff [3,4,5,6], Ellen A. Breedveld[3,4], Wendy Noort[3,4], Carla Offringa[3,4], Frank W. Bloemers[4,7], Michel van Weeghel[8], Bauke V. Schomakers[8], Pedro Coelho[9,10,11], Jelle J. Posthuma[7,12], Eleonora Aronica[11], W Joost Wiersinga[1,2, 13], Michèle van Vugt[2,14]*†, Rob C.I. Wüst[3,4]*†

† Authors contributed equally, * Corresponding authors

[1] Amsterdam UMC location University of Amsterdam, Center for Experimental and Molecular Medicine, Meibergdreef 9, Amsterdam, the Netherlands

[2] Amsterdam Institute for Infection and Immunity, Infectious diseases, Amsterdam, Netherlands

[3] Department of Human Movement Sciences, Faculty of Behavioral and Movement Sciences, Vrije Universiteit Amsterdam, Amsterdam, the Netherlands

[4] Amsterdam Movement Sciences, Amsterdam, the Netherlands

[5] Amsterdam UMC location Vrije Universiteit Amsterdam, Department of Physiology, De Boelelaan 1117, Amsterdam, the Netherlands

[6] Amsterdam Cardiovascular Sciences, Amsterdam, the Netherlands

[7] Amsterdam UMC location University of Amsterdam, Department of Trauma Surgery, Meibergdreef 9, Amsterdam, the Netherlands

[8] Amsterdam UMC location University of Amsterdam, Laboratory Genetic Metabolic Diseases, Core Facility Metabolomics, Meibergdreef 9, Amsterdam, the Netherlands

[9] Serviço de Neurologia, Departamento de Neurociências e Saúde Mental, Hospital de Santa Maria, CHULN, Lisbon, Portugal

[10] Centro de Estudos Egas Moniz, Faculdade de Medicina, University of Lisbon, Lisbon, Portugal

[11] Amsterdam UMC location University of Amsterdam, Department of (Neuro)pathology, Amsterdam Neuroscience, Meibergdreef 9, Amsterdam, the Netherlands

[12] Flevoziekenhuis, Division of Surgery, Hospitaalweg 1, Almere, the Netherlands

[13] Amsterdam UMC location University of Amsterdam, Division of Infectious Diseases, Department of Internal Medicine, Meibergdreef 9, Amsterdam, the Netherlands

[14] Amsterdam UMC location University of Amsterdam, Division of Infectious Diseases, Tropical Medicine, Department of Medicine, Meibergdreef 9, Amsterdam, the Netherlands

Supplemental Figures

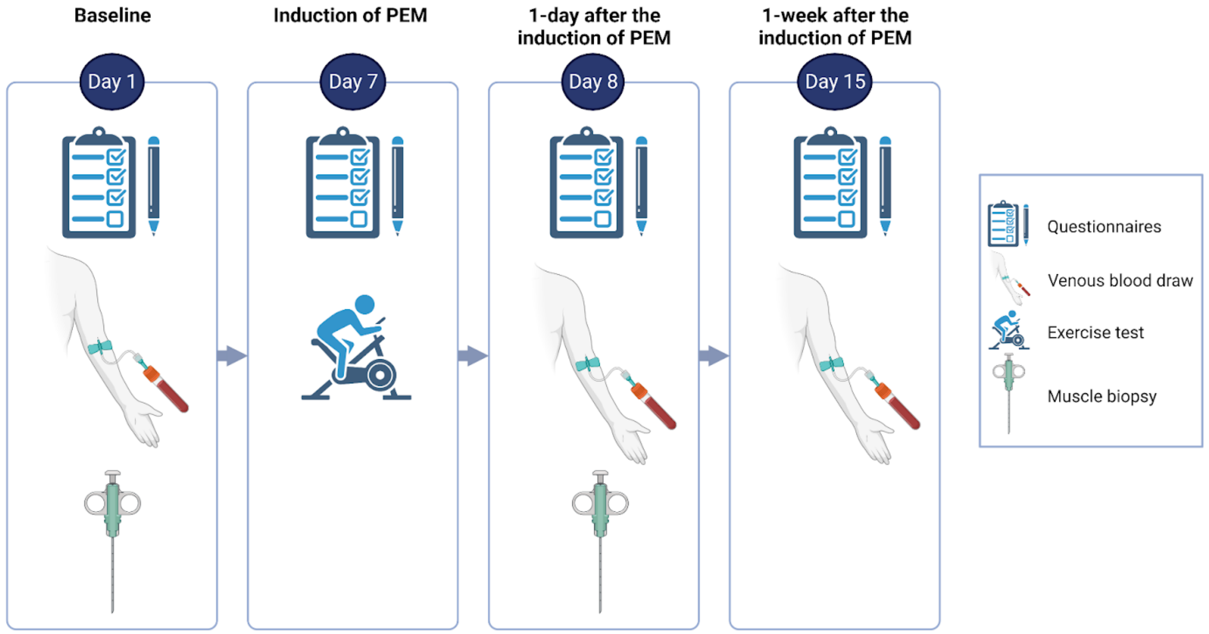

Supplemental Figure 1. Schematic overview of the study. In total, 25 patients with long COVID and 21 healthy controls were included. At baseline (day 1), venous blood in the fasted state, and a muscle biopsy of the vastus lateralis muscle was taken, alongside questionnaires (see text) about long COVID symptomatology. Six days later (day 7), a cardiopulmonary exercise test (CPET) was performed to evaluate exercise tolerance, and to induce post-exertional malaise in patients with long COVID. One day later, fasted venous blood and another skeletal muscle biopsy from the vastus lateralis muscle was taken, and questionnaires confirmed the worsening of long COVID symptoms, typical for post-exertional malaise. On day 15, 8 days after the CPET, another fasted venous blood sample was drawn, and questionnaires were filled in. During the entire study period, participants wore an Actigraph to register the total steps taken. Abbreviations: PEM; post-exertional malaise.

This image was created with BioRender (full license).

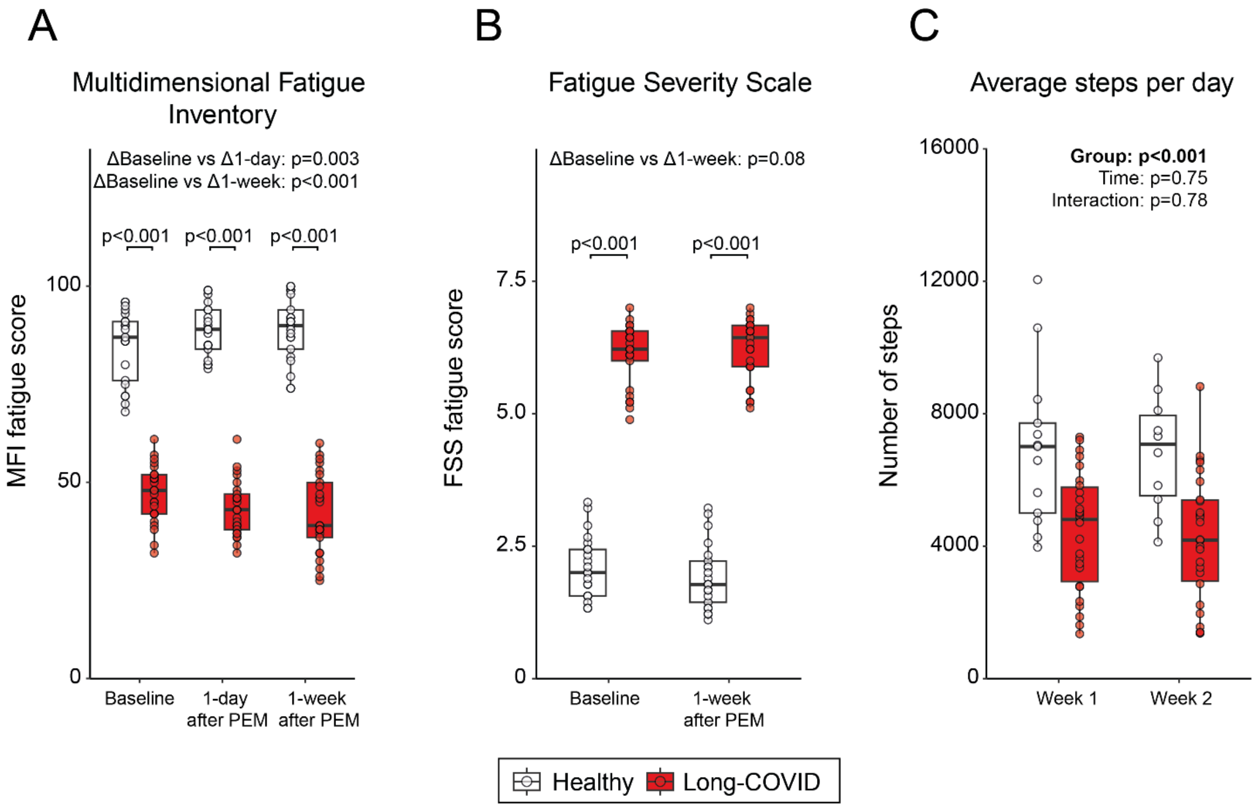

Supplemental Figure 2. Fatigue severity scores and physical activity in long COVID patients after induction of post-exertional malaise. Fatigue scores, measured with the Multidimensional Fatigue Inventory (**A**, n=25 long COVID, n=21 healthy) and the Fatigue Severity Scale (**B**) questionnaires were significantly worse in long COVID patients (n=25) compared to controls (n=21) and further worsened upon induction of post-exertional malaise for the Multidimensional Fatigue Inventory. **C**: Average step count per day at both the first week (baseline) and the second week (after the induction of PEM) was lower in patients (n=25) compared to healthy controls (n=15). Continuous nonparametric data were analyzed using a two-sided Mann–Whitney U test (panel A-B). Continuous nonparametric longitudinal data (panel A-B) were conducted by comparing the delta (Δ) between the time points using a two-sided Mann–Whitney U test and t-test. Continuous parametric longitudinal data (panel C) were analyzed with a generalized linear mixed model with a two-sided ANOVA. *p<0.05; **p<0.001. Abbreviations: PEM; post-exertional malaise. Box plots show the median (centerline), the first and third quartiles (the lower and upper bound of the box) and the whiskers show the 1.5x interquartile range. Source data are provided in the Source Data File.

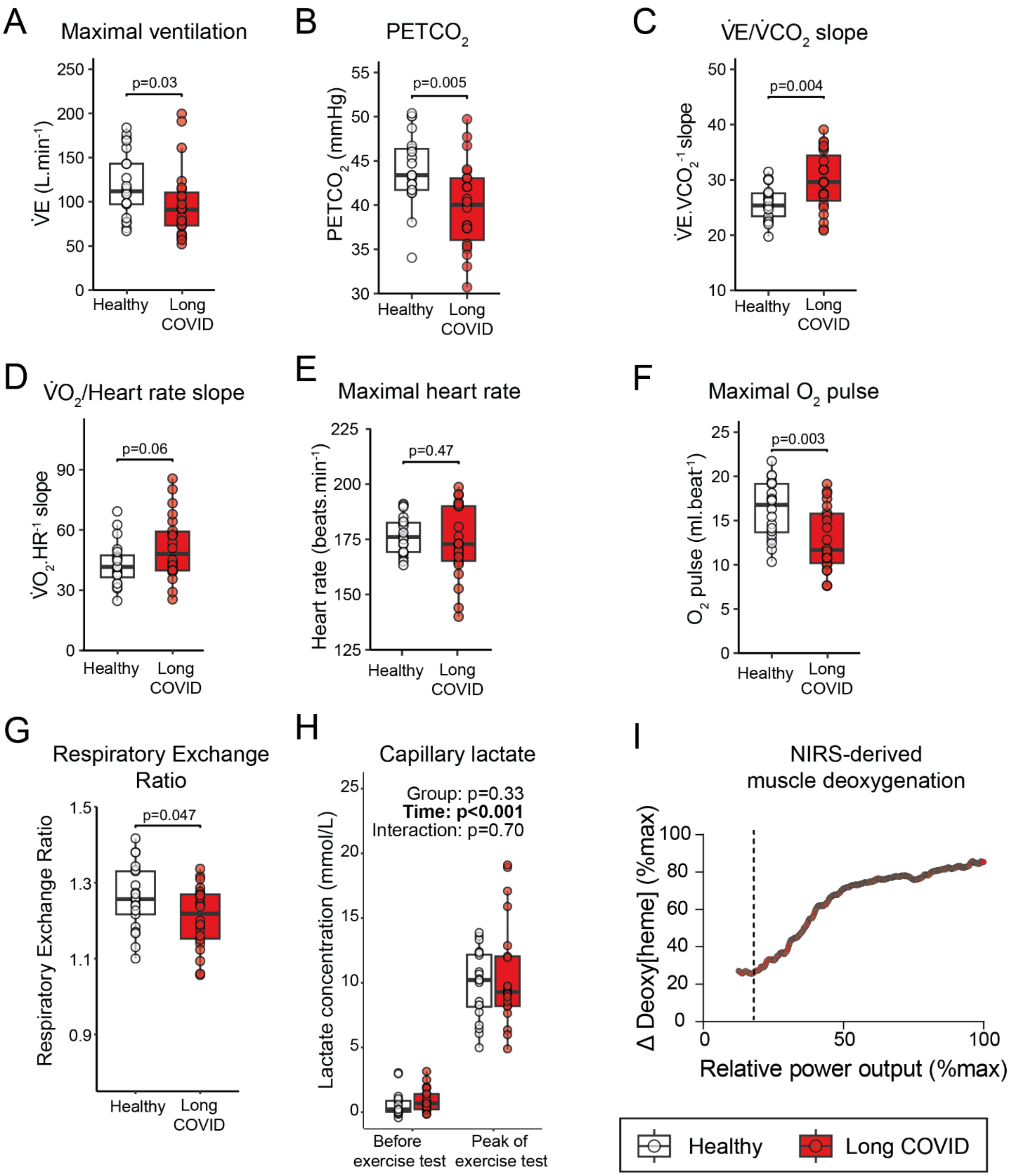

Supplemental Figure 3. Physiological measurements determined during exercise testing in healthy controls and long COVID. The maximal ventilation **(A)**, maximal end-tidal CO_2 tensions **(B)**, the slope of the relationship between ventilation and

carbon dioxide output **(C)**, the slope of the relationship between oxygen uptake and heart rate **(D)**, maximal heart rate **(E)** and maximal O_2 pulse **(F)**. The respiratory exchange ratio was above 1.10 for almost all participants **(G)**. Panel **H** shows capillary lactate before and at the peak of the exercise test. Panel **I** shows the near-infrared spectroscopy-derived muscle deoxygenation responses to ramp incremental exercise in a representative participant. Panel A-C, G; Long COVID; n=23, Healthy; n=21, panel D-F; Long COVID; n=22, Healthy; n=20, panel G; Long COVID; n=21, Healthy; n=20. Continuous parametric data were analyzed using a two-sided t-test and two-sided non-parametric with a Mann–Whitney U test (panel A-G). Continuous parametric longitudinal data (panel H) were analyzed with a generalized linear mixed model with a two-sided ANOVA. ns; non-significant, $^{*}p<0.05$. Abbreviations: $PETCO_2$; end-tidal CO_2 tension, $\dot{V}E$; minute ventilation, $\dot{V}CO_2$; pulmonary carbon dioxide output, $\dot{V}O_2$; pulmonary oxygen uptake. Box plots show the median (centerline), the first and third quartiles (the lower and upper bound of the box) and the whiskers show the 1.5x interquartile range. Source data are provided in the Source Data File.

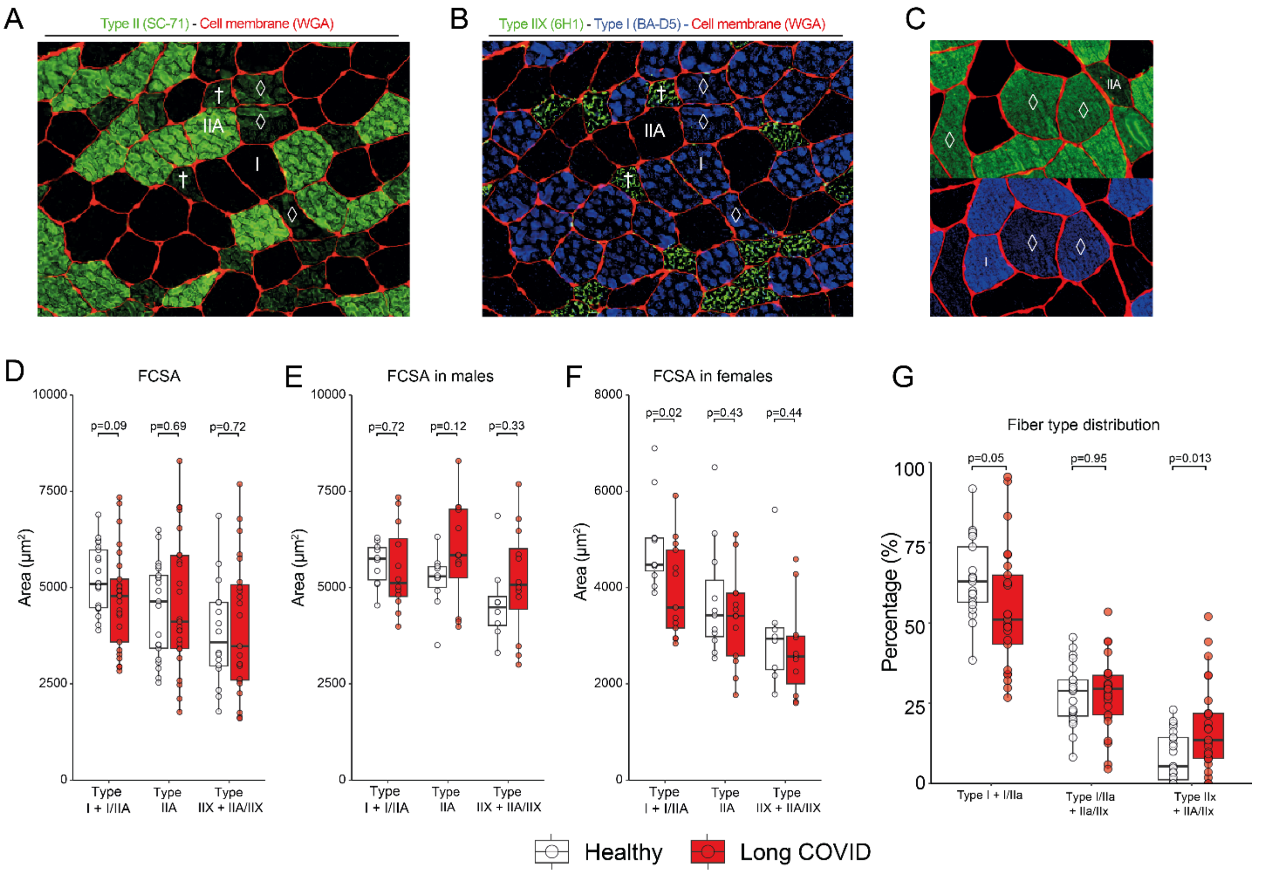

Supplemental Figure 4. Fiber types and cross-sectional area distribution in long COVID patients and healthy controls. Panels **A**-**C** show a typical example of immunofluorescence analysis of myosin heavy chain (MHC) expression in a 10-µm cross-section of the vastus lateralis of long COVID patients with a high proportion of hybrid fibers. In panels **D-F,** we show that there is no difference in the fiber cross-sectional area between healthy controls (n=21) and long COVID patients (n=25), although the type **I** fiber cross-sectional area is smaller in females with long COVID compared to healthy female controls **(F)**. **G:** Patients with long COVID (n=25) had a higher percentage of glycolytic type IIx skeletal muscle fibers compared to healthy controls (n=21). Continuous parametric data were analyzed using a two-sided t-test and non-parametric data with a two-sided Mann–Whitney U (panel D-F). Abbreviations: IIA; type IIA skeletal muscle fiber, IIX; type IIX skeletal muscle fiber, ◊; I/IIA hybrid skeletal muscle fibers, †; IIA/IIX hybrid skeletal muscle fiber, FCSA; fiber cross-sectional area. Box plots show the median (centerline), the first and third quartiles (the lower and upper bound of the box) and the whiskers show the 1.5x interquartile range. Source data are provided in the Source Data File.

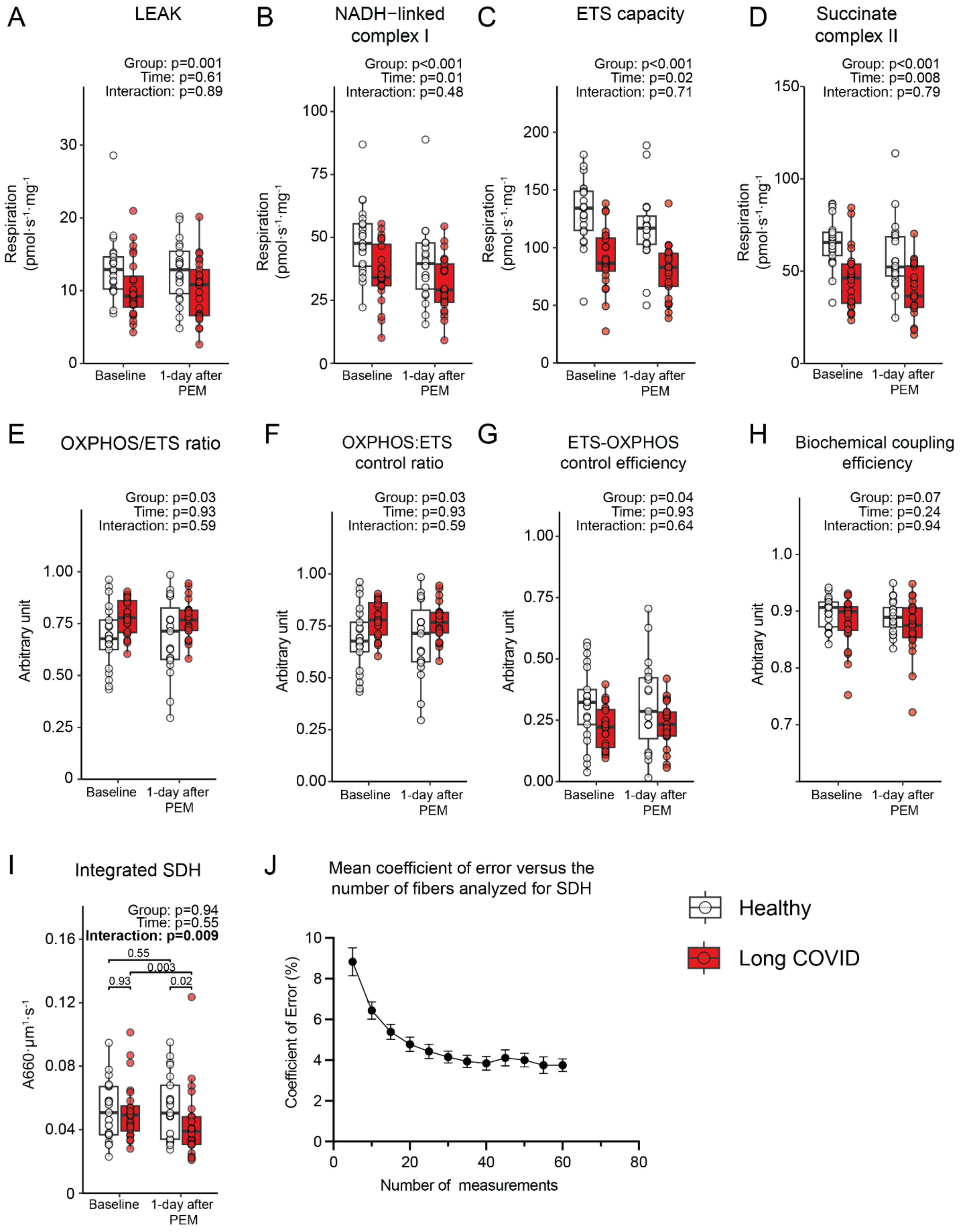
A
LEAK
Group: p=0.001
Time: p=0.61
Interaction: p=0.89
Respiration (pmol·s⁻¹·mg⁻¹)
Baseline
1-day after PEM
B
NADH−linked complex I
Group: p<0.001
Time: p=0.01
Interaction: p=0.48
C
ETS capacity
Group: p<0.001
Time: p=0.02
Interaction: p=0.71
D
Succinate complex II
Group: p<0.001
Time: p=0.008
Interaction: p=0.79
E
OXPHOS/ETS ratio
Group: p=0.03
Time: p=0.93
Interaction: p=0.59
Arbitrary unit
F
OXPHOS:ETS control ratio
Group: p=0.03
Time: p=0.93
Interaction: p=0.59
G
ETS-OXPHOS control efficiency
Group: p=0.04
Time: p=0.93
Interaction: p=0.64
H
Biochemical coupling efficiency
Group: p=0.07
Time: p=0.24
Interaction: p=0.94
I
Integrated SDH
Group: p=0.94
Time: p=0.55
Interaction: p=0.009
0.55
0.003
0.93
0.02
A660·µm⁻¹·s⁻¹
J
Mean coefficient of error versus the number of fibers analyzed for SDH
Coefficient of Error (%)
Number of measurements
Healthy
Long COVID

Supplemental Figure 5. Skeletal muscle mitochondrial respiration in patients with long COVID and healthy controls. Leak respiration (**A**), mitochondrial respiration with NADH-linked substrates (glutamate, pyruvate and malate, **B**), electron transport capacity (ETS, with succinate and FCCP, **C**) and uncoupled succinate-linked respiration (with rotenone, **D**) were lower in patients with long COVID compared to healthy controls, but no effect of PEM was observed. The ratio of oxidative phosphorylation (OXPHOS) capacity to ETS capacity **(E)**, the OXPHOS:ETS control ratio **(F)** and the ETS-OXPHOS control efficiency **(G)** showed significant differences between long-COVID and healthy controls. The biochemical coupling **(H)** efficiency showed no difference between healthy control and long COVID. Integrated succinate dehydrogenase (SDH) activity (i.e. fiber cross-sectional area x SDH activity**, I**), indicative of the amount of oxidative enzyme per fiber, showed a significant interaction effect. Mean (± SD) coefficient of error versus the number of fibers analyzed for SDH activity **(J)**. This analysis indicated that a minimum of 40 fibers per participant was required to reduce the coefficient of error in the analysis of SDH activity. All panels consisted of 25 long COVID patients and 21 healthy controls. Continuous parametric longitudinal data (panel A-I) were analyzed with a generalized linear mixed model with a two-sided ANOVA. Abbreviations: PEM; post-exertional malaise. SDH; succinate dehydrogenase activity. Box plots show the median (centerline), the first and third quartiles (the lower and upper bound of the box) and the whiskers show the 1.5x interquartile range. Source data are provided in the Source Data File.

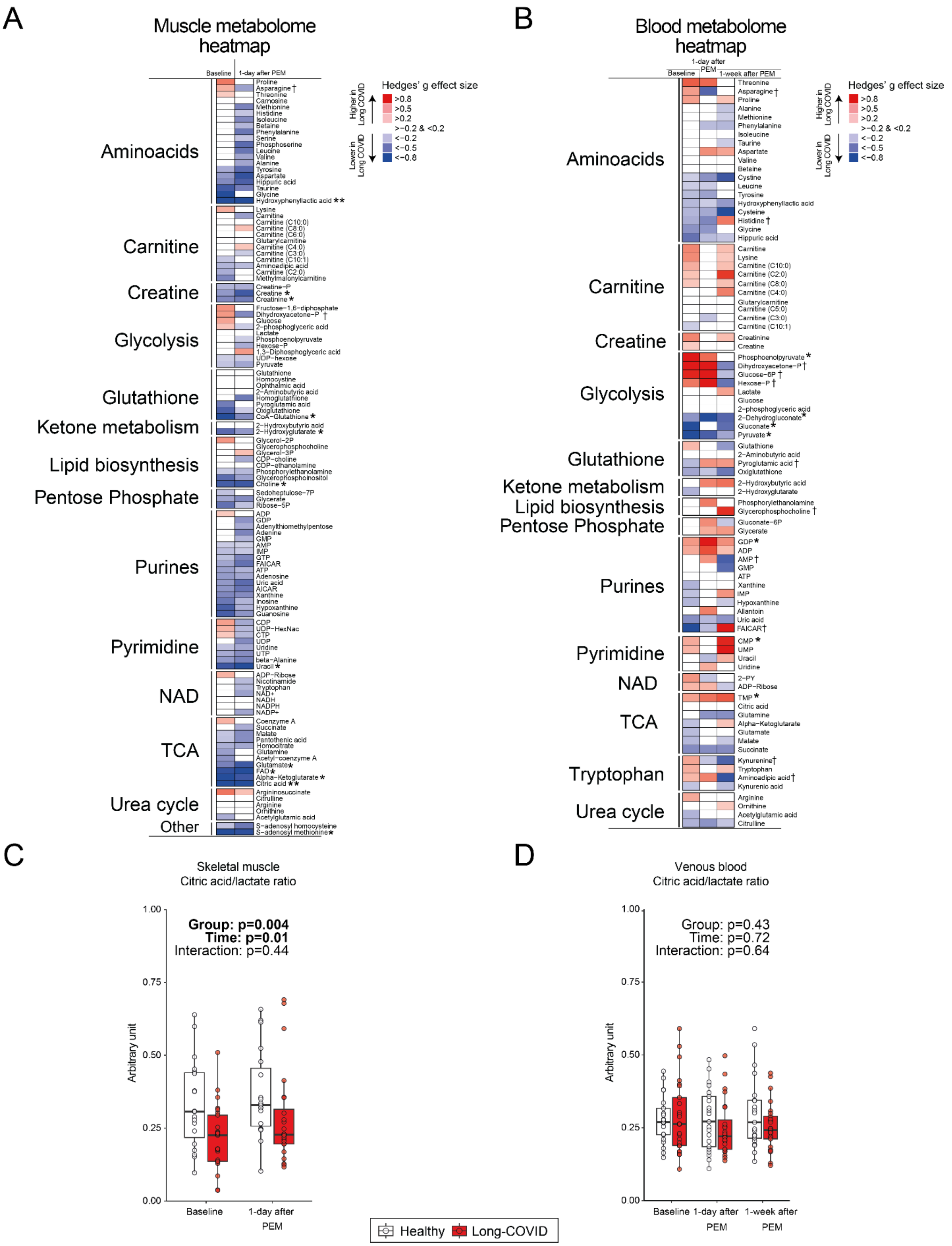
A
Muscle metabolome heatmap
Baseline
1-day after PEM
Aminoacids
Carnitine
Creatine
Glycolysis
Glutathione
Ketone metabolism
Lipid biosynthesis
Pentose Phosphate
Purines
Pyrimidine
NAD
TCA
Urea cycle
Other
Hedges' g effect size
Higher in Long COVID
Lower in Long COVID
>0.8
>0.5
>0.2
>−0.2 & <0.2
<−0.2
<−0.5
<−0.8
B
Blood metabolome heatmap
Baseline
1-day after PEM
1-week after PEM
Aminoacids
Carnitine
Creatine
Glycolysis
Glutathione
Ketone metabolism
Lipid biosynthesis
Pentose Phosphate
Purines
Pyrimidine
NAD
TCA
Tryptophan
Urea cycle
C
Skeletal muscle
Citric acid/lactate ratio
Group: p=0.004
Time: p=0.01
Interaction: p=0.44
Arbitrary unit
1.00
0.75
0.50
0.25
0.00
Baseline
1-day after PEM
D
Venous blood
Citric acid/lactate ratio
Group: p=0.43
Time: p=0.72
Interaction: p=0.64
Arbitrary unit
1.00
0.75
0.50
0.25
0.00
Baseline
1-day after PEM
1-week after PEM
Healthy
Long-COVID

Supplemental Figure 6. Skeletal and venous metabolome before and after induction of post-exertional malaise in long COVID patients. A: Heatmap depicting Hedges' g effect size of muscle metabolites and the associated pathways comparing long COVID patients (n=25) with healthy controls (n=19) at both timepoints. **B:** Heatmap depicting Hedges' g effect size of venous blood metabolites and the associated pathways comparing long COVID (n=25) patients with healthy controls (n=21). Highlighted is the citric acid/lactate ratio in skeletal muscle **(C)** and in venous blood **(D)**. Continuous parametric longitudinal data (panel A-D) were analyzed with a generalized linear mixed model with a two-sided ANOVA. Effect sizes (panel A-B) were calculated with Hedges `g. Abbreviations: NAD; Nicotinamide adenine dinucleotide. PEM; post-exertional malaise, TCA; tricarboxylic acid cycle. Significance of long COVID compared to healthy controls (Group) is shown with *p<0.05; **p<0.001. A significant interaction term is shown with: "†", depicting a p<0.05. Box plots show the median (centerline), the first and third quartiles (the lower and upper bound of the box) and the whiskers show the 1.5x interquartile range. Source data are provided in the Source Data File.

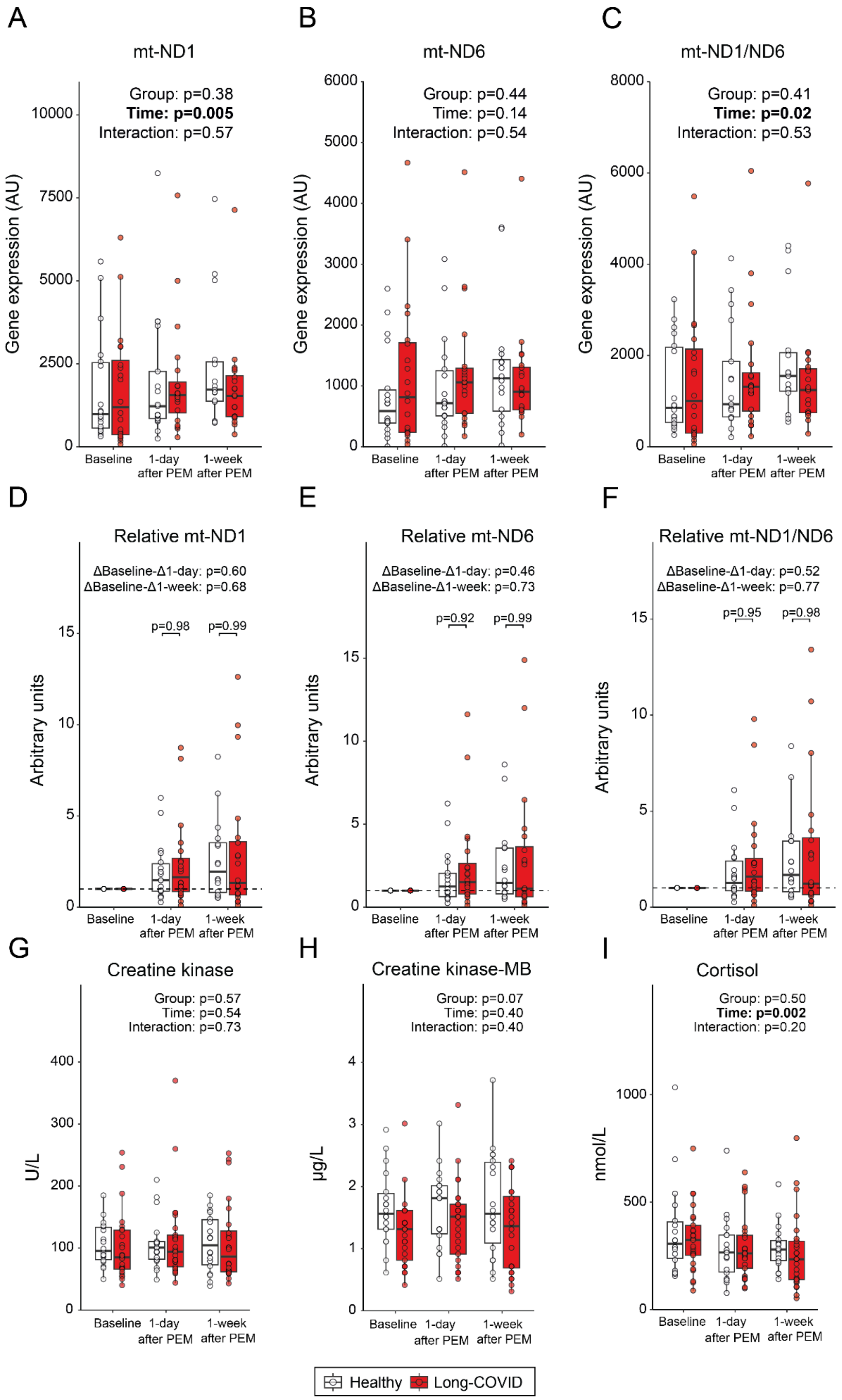

A
mt-ND1
Group: p=0.38
Time: p=0.005
Interaction: p=0.57
Gene expression (AU)
B
mt-ND6
Group: p=0.44
Time: p=0.14
Interaction: p=0.54
C
mt-ND1/ND6
Group: p=0.41
Time: p=0.02
Interaction: p=0.53
Baseline
1-day after PEM
1-week after PEM
D
Relative mt-ND1
ΔBaseline-Δ1-day: p=0.60
ΔBaseline-Δ1-week: p=0.68
p=0.98
p=0.99
Arbitrary units
E
Relative mt-ND6
ΔBaseline-Δ1-day: p=0.46
ΔBaseline-Δ1-week: p=0.73
p=0.92
p=0.99
F
Relative mt-ND1/ND6
ΔBaseline-Δ1-day: p=0.52
ΔBaseline-Δ1-week: p=0.77
p=0.95
p=0.98
G
Creatine kinase
Group: p=0.57
Time: p=0.54
Interaction: p=0.73
U/L
H
Creatine kinase-MB
Group: p=0.07
Time: p=0.40
Interaction: p=0.40
μg/L
I
Cortisol
Group: p=0.50
Time: p=0.002
Interaction: p=0.20
nmol/L
Healthy
Long-COVID

Supplemental Figure 7. Plasma mitochondrial DNA and circulating markers of tissue damage and stress. The gene expression of circulating mtDNA (△△Ct of ND1 and ND6) normalized to 18S **(A-C)** and the fold change as compared to the baseline measurement **(**△△Ct; **D-F)**. Panel A-F: Long COVID; n=21, Healthy; n=21. Creatine kinase **(G)**, creatine kinase-MB **(H)** as measured in venous blood were not significantly different. Cortisol **(I)** showed a significant difference over time with no difference between healthy controls and long COVID patients. Adjusting cortisol concentration for the minutes since waking up did not change results. Panel G-I; Long COVID; n=25, Healthy; n=18. The dotted lines in panels D-F represents the baseline value of 1. Continuous parametric longitudinal data (panel A-C, G-I) were analyzed with a generalized linear mixed model with a two-sided ANOVA. Continuous nonparametric longitudinal data (panel D-F) were conducted by comparing the delta (Δ) between the time points using a two-sided Mann–Whitney U test and t-test. Abbreviations: mt-ND1; mitochondrially-encoded NADH-ubiquinone oxidoreductase chain 1, mt-ND6; mitochondrially-encoded NADH-ubiquinone oxidoreductase chain 6, PEM; post-exertional malaise. Box plots show the median (centerline), the first and third quartiles (the lower and upper bound of the box) and the whiskers show the 1.5x interquartile range. Source data are provided in the Source Data File.

STROBE Statement—Checklist of items that should be included in reports of ***cohort studies***

	Item No	Recommendation	Page No
**Title and abstract**	1	(*a*) Indicate the study's design with a commonly used term in the title or the abstract<br>(*b*) Provide in the abstract an informative and balanced summary of what was done and what was found	1+2
**Introduction**			
Background/rationale	2	Explain the scientific background and rationale for the investigation being reported	3
Objectives	3	State specific objectives, including any prespecified hypotheses	3
**Methods**			
Study design	4	Present key elements of study design early in the paper	11-13
Setting	5	Describe the setting, locations, and relevant dates, including periods of recruitment, exposure, follow-up, and data collection	11-13
Participants	6	(a) Give the eligibility criteria, and the sources and methods of selection of participants. Describe methods of follow-up<br>(b) For matched studies, give matching criteria and number of exposed and unexposed	11
Variables	7	Clearly define all outcomes, exposures, predictors, potential confounders, and effect modifiers. Give diagnostic criteria, if applicable	11-16
Data sources/ measurement	8*	For each variable of interest, give sources of data and details of methods of assessment (measurement). Describe comparability of assessment methods if there is more than one group	11-16
Bias	9	Describe any efforts to address potential sources of bias	3, 11-16
Study size	10	Explain how the study size was arrived at	3, 11
Quantitative variables	11	Explain how quantitative variables were handled in the analyses. If applicable, describe which groupings were chosen and why	16
Statistical methods	12	(a) Describe all statistical methods, including those used to control for confounding<br>(b) Describe any methods used to examine subgroups and interactions<br>(c) Explain how missing data were addressed<br>(d) If applicable, explain how loss to follow-up was addressed<br>(e) Describe any sensitivity analyses	16
**Results**			
Participants	13*	(a) Report numbers of individuals at each stage of study—eg numbers potentially eligible, examined for eligibility, confirmed eligible, included in the study, completing follow-up, and analysed<br>(b) Give reasons for non-participation at each stage<br>(c) Consider use of a flow diagram	3-7 Captions of figures
Descriptive data	14*	(a) Give characteristics of study participants (eg demographic, clinical, social) and information on exposures and potential confounders<br>(b) Indicate number of participants with missing data for each variable of interest	3-7

		(c) Summarise follow-up time (eg, average and total amount)	
Outcome data	15*	Report numbers of outcome events or summary measures over time	3-7
Main results	16	(a) Give unadjusted estimates and, if applicable, confounder-adjusted estimates and their precision (eg, 95% confidence interval). Make clear which confounders were adjusted for and why they were included<br>(b) Report category boundaries when continuous variables were categorized<br>(c) If relevant, consider translating estimates of relative risk into absolute risk for a meaningful time period	3-7
Other analyses	17	Report other analyses done—eg analyses of subgroups and interactions, and sensitivity analyses	3-7
**Discussion**			
Key results	18	Summarise key results with reference to study objectives	3-10
Limitations	19	Discuss limitations of the study, taking into account sources of potential bias or imprecision. Discuss both direction and magnitude of any potential bias	3-10
Interpretation	20	Give a cautious overall interpretation of results considering objectives, limitations, multiplicity of analyses, results from similar studies, and other relevant evidence	3-10
Generalisability	21	Discuss the generalisability (external validity) of the study results	3-10
**Other information**			
Funding	22	Give the source of funding and the role of the funders for the present study and, if applicable, for the original study on which the present article is based	1

*Give information separately for exposed and unexposed groups.

Note: An Explanation and Elaboration article discusses each checklist item and gives methodological background and published examples of transparent reporting. The STROBE checklist is best used in conjunction with this article (freely available on the Web sites of PLoS Medicine at http://www.plosmedicine.org/, Annals of Internal Medicine at http://www.annals.org/, and Epidemiology at http://www.epidem.com/). Information on the STROBE Initiative is available at http://www.strobe-statement.org.
