# Supplementary material for: Muscle abnormalities worsen after post-exertional malaise in long COVID
Source: Nat Commun. 2024 Jan 4;15:17. doi: 10.1038/s41467-023-44432-3 (PMC10766651; doi:10.1038/s41467-023-44432-3)
Supplement: Supplementary file 3 — Reporting Summary [file 41467_2023_44432_MOESM3_ESM.pdf]

Corresponding author(s): Rob Wüst

Last updated by author(s): Nov 23, 2023

Reporting Summary

Nature Portfolio wishes to improve the reproducibility of the work that we publish. This form provides structure for consistency and transparency in reporting. For further information on Nature Portfolio policies, see our Editorial Policies and the Editorial Policy Checklist.

Statistics

For all statistical analyses, confirm that the following items are present in the figure legend, table legend, main text, or Methods section.

n/a	Confirmed	
☐	☒	The exact sample size (*n*) for each experimental group/condition, given as a discrete number and unit of measurement
☐	☒	A statement on whether measurements were taken from distinct samples or whether the same sample was measured repeatedly
☐	☒	The statistical test(s) used AND whether they are one- or two-sided<br>*Only common tests should be described solely by name; describe more complex techniques in the Methods section.*
☐	☒	A description of all covariates tested
☐	☒	A description of any assumptions or corrections, such as tests of normality and adjustment for multiple comparisons
☐	☒	A full description of the statistical parameters including central tendency (e.g. means) or other basic estimates (e.g. regression coefficient) AND variation (e.g. standard deviation) or associated estimates of uncertainty (e.g. confidence intervals)
☐	☒	For null hypothesis testing, the test statistic (e.g. *F*, *t*, *r*) with confidence intervals, effect sizes, degrees of freedom and *P* value noted<br>*Give P values as exact values whenever suitable.*
☒	☐	For Bayesian analysis, information on the choice of priors and Markov chain Monte Carlo settings
☒	☐	For hierarchical and complex designs, identification of the appropriate level for tests and full reporting of outcomes
☐	☒	Estimates of effect sizes (e.g. Cohen's *d*, Pearson's *r*), indicating how they were calculated

Our web collection on statistics for biologists contains articles on many of the points above.

Software and code

Policy information about availability of computer code

Data collection	Exercise test: Lode Excalibur Sport, Lode, Groningen, The Netherlands<br>Pulmonary gas exchange: Cosmed Quark CPET; Cosmed, Rome, Italy.<br>Lactate measurement: Lactate Pro 2 LT-1730, ARKRAY Ltd., United Kingdom.<br>Step counter: Actigraph wGT3X-BT<br>NIRS device: Portamon, Artinis Medical Systems, Arnhem, The Netherlands<br>Mitochondrial respiration data collection software: DatLab Version 7.4.0.4 (Ororoboros Instruments, Innsbruck, AT)<br>Metabolomics was collected using using Bruker TASQ software version 2.1.22.3.<br>Clinical data was collected in CASTOR EDC
Data analysis	Data analysis was performed using packages and functions in R version 4.0.3. The following R packages were used for data analysis and visualizations: readxl 1.4.3, tableone 0.13.2, car 3.1-2, nortest 1.0-4, MASS 7.3-58.1, nlme 3.1-160, circlize 0.4.15, rstatix 0.7.2, mmeans 4.2.3, ComplexHeatmap 2.14.0, cocor 1.1-4, ggplot2 3.4.2, ggsignif 0.6.4, ggpubr 0.6.0.<br><br>Fiber type composition was performed with ImageJ and Sandia Matlab AnalysiS Hierarchy (SMASH) Toolbox (version 1.0) in Matlab (version 2022a).<br><br>R code used to perform analysis and generate figures along are made available at https://zenodo.org/doi/10.5281/zenodo.10171056<br>All figure panels were assembled using Adobe Illustrator (version 27.2; https://www.adobe.com/products/illustrator.html).

For manuscripts utilizing custom algorithms or software that are central to the research but not yet described in published literature, software must be made available to editors and reviewers. We strongly encourage code deposition in a community repository (e.g. GitHub). See the Nature Portfolio guidelines for submitting code & software for further information.

Data

Policy information about availability of data

All manuscripts must include a data availability statement. This statement should provide the following information, where applicable:

- Accession codes, unique identifiers, or web links for publicly available datasets
- A description of any restrictions on data availability
- For clinical datasets or third party data, please ensure that the statement adheres to our policy

Metabolomic source data is available on: https://doi.org/10.48338/VU01-KPABVO.
Source data for all figures can be found in the file Source Data.
All metabolomics will also be uploaded on MetaboLights (EU Server)

Research involving human participants, their data, or biological material

Policy information about studies with human participants or human data. See also policy information about sex, gender (identity/presentation), and sexual orientation and race, ethnicity and racism.

Reporting on sex and gender	Sex is reported for all participants. Sex of participants was determined based on self-report. Patients were enrolled based on having long COVID. All long COVID were age and sex matched with the control group. There was no sex or gender analysis. The medical ethics committee of the Amsterdam UMC approved the study (NL78394.018.21)
Reporting on race, ethnicity, or other socially relevant groupings	There was no reporting on race, ethnicity or other socially relevant grouping.
Population characteristics	Long COVID patients were diagnosed by two experienced clinicians for long COVID symptomology and exclusion of potential differential diagnoses. All long COVID patients were diagnosed with post-exertional malaise (PEM) by the DSQ-PEM (1), had a minimum period of long COVID-related symptoms of six months, and were between 18 and 65 years old. Questionnaires regarding fatigue, fatigue severity score (FSS)(2), multidimensional fatigue inventory (MFI)(3) and DSQ-PEM were obtained throughout the study period. None of the included participants were admitted to the hospital during acute SARS-CoV-2 infection and were healthy prior to NAAT or serology-proven SARS-CoV-2 infection. Exclusion criteria were a medical history of cardiovascular/pulmonary disease, diabetes mellitus, or concurrent treatment with metabolism or coagulant-altering drugs during the study period (statins, corticosteroids, SGLT2 inhibitors, GLP1 receptor agonists, platelet aggregation blockers and any anticoagulants). None of the healthy controls had residual symptoms after the SARS-CoV-2 infection. One long COVID patient was excluded due to a recent SARS-CoV-2 re-infection (<7 days). Healthy controls withdrew because of the invasive nature of the protocol (n=2), symptoms related to a burn-out (n=1), and a novel diagnosis of uncontrolled hypertension (n=1).
Recruitment	Long COVID patients and healthy controls were recruited from the Amsterdam UMC post-covid clinic. Long COVID patients were diagnosed by two experienced clinicians for long COVID symptomology and exclusion of potential differential diagnoses. All long COVID patients were diagnosed with post-exertional malaise (PEM) by the DSQ-PEM, had a minimum period of long COVID-related symptoms of six months, and were between 18 and 65 years old.<br><br>None of the included participants were admitted to the hospital during acute SARS-CoV-2 infection and were healthy prior to NAAT or serology-proven SARS-CoV-2 infection. Exclusion criteria were a medical history of cardiovascular/pulmonary disease, diabetes mellitus, or concurrent treatment with metabolism or coagulant-altering drugs during the study period (statins, corticosteroids, SGLT2 inhibitors, GLP1 receptor agonists, platelet aggregation blockers and any anticoagulants). None of the healthy controls had residual symptoms after the SARS-CoV-2 infection<br><br>Our inclusion from the post-covid clinic may have let to a more severe ill population as they are the ones to seek assistance of healthcare professionals.
Ethics oversight	The medical ethics committee of the Amsterdam UMC approved the study (NL78394.018.21)

Note that full information on the approval of the study protocol must also be provided in the manuscript.

Field-specific reporting

Please select the one below that is the best fit for your research. If you are not sure, read the appropriate sections before making your selection.

☒ Life sciences ☐ Behavioural & social sciences ☐ Ecological, evolutionary & environmental sciences

For a reference copy of the document with all sections, see nature.com/documents/nr-reporting-summary-flat.pdf

Life sciences study design

All studies must disclose on these points even when the disclosure is negative.

Sample size	A reduction in exercise capacity is a prerequisite for the identification of post-exertional fatigue. As the reported time-course of reductions in exercise capacity with post-exertional malaise syndrome is highly similar, the data of Snell et al. (2013) can be utilized to determine the required sample size for this project. Snell et al. (2013) reported a large effect size for differences in exercise capacity between groups (Cohen's d = 1.1). Hence, to assess for the hypothesized effect size of 1.1 with a two-tailed alpha level of 0.05 and a β of 0.95, with a case-control ratio of 1, a group size of 19 would be required.<br>We anticipate that the heterogeneity of our patient population is larger, and that not all patients will undergo all testing. Therefore, we conservatively assess that a group size of 25 will provide enough statistical and clinical power
Data exclusions	- Two participants mitochondrial respiration were excluded due to not passing the quality control<br>- Four participants metabolomics data were excluded due to not passing the quality control<br>No other data were excluded
Replication	All exercise data were not replicated as this would introduce additional post-exertional malaise. Analysis of blood was not repeated but included various control samples. Muscle sections stainings were performed on multiple sections of muscle and we only show typical examples within the manuscript. Furthermore, by utilizing longitudonal samples we are confident we show stable and persistent within this manuscript.
Randomization	There was no randomization in the study procedure. All participants were age and sex matched and followed the same study procedure.
Blinding	Investigators were not blinded for the study as this was an observational study.

Reporting for specific materials, systems and methods

We require information from authors about some types of materials, experimental systems and methods used in many studies. Here, indicate whether each material, system or method listed is relevant to your study. If you are not sure if a list item applies to your research, read the appropriate section before selecting a response.

Materials & experimental systems

n/a	Involved in the study	
☐	☒	Antibodies
☒	☐	Eukaryotic cell lines
☒	☐	Palaeontology and archaeology
☒	☐	Animals and other organisms
☐	☒	Clinical data
☒	☐	Dual use research of concern
☒	☐	Plants

Methods

n/a	Involved in the study	
☒	☐	ChIP-seq
☒	☐	Flow cytometry
☒	☐	MRI-based neuroimaging

Antibodies

Antibodies used

See supplement for full table:
Muscle fiber type (Antibody, dilution, vendor):
BA-D5 (MHC-I), 1ug/ml, DSHB; SC-71 (MHC-IIA), 1ug/ml, DSHB; 6H1 (MHC-IIX) 5ug/ml, DSHB (60 min); WGA 350, 1:25, TMO W11263 (30 min); MHCI (BA-D5), 1ug/ml, DSHB; MHC IIA (SC-71), 1ug/ml, DSHB; MHC IIX (6H1), 5ug/ml, DSHB (60 min); WGA 350, 1:25, TMO W11263 (30 min)

Muscle fiber type (Secondary antibody (dilution), vendor):
Goat-anti mouse IgG2b 555, 1:1000; Invitrogen A21147; Goat anti-mouse IgG1 647, 1:1000; Invitrogen A21240; Goat anti-mouse IgM 488, 1:1000; Invitrogen A21042 (60 min)

RRRID Muscle fiber type
AB_2235587AB_2147165; AB_1157897;
AB_2535783; AB_2535809; AB_2535711

Amyloid-containing deposits:
Thioflavin T (50µM stock), 1:10, Sigma-Aldrich, (30 minutes), WGA 555, 1:25, TMO W32464 (30 min)

Endothelial cells:
CD31, IgG1 mouse, 1:50, Abcam ab9498, (overnight incubation), WGA 350, 1:25, TMO W11263 (30 min).
Secondary antibody: Goat anti-mouse IgG1 647, 1:200, Invitrogen A21240, (60 minutes).
RRID: AB_726362

Endothelial cells:

Biotinylated UEA-1, 1:100, B-1065, Vector Laboratories, (30 minutes), AB_2336766
Vectastain Elite ABC Kit PK-6100, Vector Laboratories (30 min);AB_2336819
ImmPACT™ AMEC Red Peroxidase Substrate SK-4285, Vector Laboratories (10 min); AB_2336519

Lymphatic vessel:
LYVE1, IgG rabbit, 1:50 Abcam 10278, (overnight incubation), WGA 350, 1:25, TMO W11263 (30 min)
Secondary antibody: Goat anti-rabbit IgG 647, 1:200, Invitrogen A32733 (60 minutes)
RRID: AB_881387

SARS-CoV-2 nucleocapsid protein:
SARS-CoV-2 Nucleocapsid Antibody, Rabbit PAb, Antigen Affinity Purified, 1:500; SinoBiological (60 min), WGA 555, 1:25, TMO W32464 (30 min),
Secondary antibody: Alexa fluor goat-anti rabbit IgG 488 1:500, Invitrogen A27034 (60 min)
RRID: AB_2892769

CD3:
SP7 (rabbit moab), 1:200, Thermo Sc. Rm-9107-S, AB_149924

CD68:
PG-M1 IgG 3, 1:200, DAKO M0876, AB_2074844

CD20:
L26 IgG 2a, 1:1000,DAKO / M0755, AB_2282030

Validation

All antibodies are commercially available and validated by the vendors. No additional validation was performed. Listed below are relevant citations. Full data can be found in table 1.

Muscle fiber type
BA-D5 (MHC-I), 1ug/ml, DSHB, (DSHB Cat# BA-D5, RRID:AB_2235587), confirmed species reactivity for humans, Schiaffino S, Gorza L, Sartore S, Saggin L, Ausoni S, Vianello M, Gundersen K, Lømo T. Three myosin heavy chain isoforms in type 2 skeletal muscle fibres. J Muscle Res Cell Motil. 1989 Jun;10(3):197-205. doi: 10.1007/BF01739810. PMID: 2547831.
SC-71 (MHC-IIA), 1ug/ml, DSHB, (DSHB Cat# SC-71, RRID:AB_2147165) ;confirmed species reactivity for humans, Schiaffino S, Gorza L, Sartore S, Saggin L, Ausoni S, Vianello M, Gundersen K, Lømo T. Three myosin heavy chain isoforms in type 2 skeletal muscle fibres. J Muscle Res Cell Motil. 1989 Jun;10(3):197-205. doi: 10.1007/BF01739810. PMID: 2547831.
6H1 (MHC-IIX) 5ug/ml, DSHB (60 min), (DSHB Cat# 6H1, RRID:AB_1157897), confirmed species reactivity for humans,
Goat-anti mouse IgG2b 555, 1:1000, Invitrogen A21147; (Thermo Fisher Scientific Cat# A-21147, RRID:AB_2535783)
Goat anti-mouse IgG1 647, 1:1000, Invitrogen A21240; (Thermo Fisher Scientific Cat# A-21240, RRID:AB_2535809)
Goat anti-mouse IgM 488, 1:1000, Invitrogen A21042; (Thermo Fisher Scientific Cat# A-21042, RRID:AB_2535711)

Amyloid-containing deposits
Thioflavin T (50µM stock), 1:10, Sigma-Aldrich, Tjernberg, L.O., et al. 1999. J. Biol. Chem.274, 12619.
WGA 555, 1:25, TMO W32464

Endothelial cells
CD31, IgG1 mouse, 1:50, Abcam ab9498, confirmed species reactivity for humans, (Abcam Cat# ab28364, RRID:AB_726362)
Goat anti-mouse IgG1 647, 1:200, Invitrogen A21240, Goat anti-Mouse IgG1 Cross-Adsorbed Secondary Antibody, Alexa Fluor™ 647 from Thermo Fisher Scientific, catalog # A-21240, RRID AB_2535809.

Endothelial cells
Biotinylated UEA-1, 1:100, B-1065, Vector Laboratories, (Vector Laboratories Cat# B-1065, RRID:AB_2336766)
Vectastain Elite ABC Kit PK-6100, Vector Laboratories, (Vector Laboratories Cat# PK-6100, RRID:AB_2336819)
ImmPACT™ AMEC Red Peroxidase Substrate SK-4285, Vector Laboratories; (Vector Laboratories Cat# SK-4285, RRID:AB_2336519)

Lymphatic vessel
LYVE1, IgG rabbit, 1:50 Abcam 10278, (Abcam Cat# ab33682, RRID:AB_881387)
Goat anti-rabbit IgG 647, 1:200, Invitrogen A32733 (Thermo Fisher Scientific Cat# A32733, RRID:AB_2633282)

SARS-CoV-2 nucleocapsid protein
SARS-CoV-2 Nucleocapsid Antibody, Rabbit PAb, Antigen Affinity Purified, 1:500; SinoBiological (Sino Biological Cat# 40143-T62, RRID:AB_2892769)
Alexa fluor goat-anti rabbit IgG 488 1:500, Invitrogen A27034

CD3
SP7 (rabbit moab), 1:200, Thermo Sc. Rm-9107-S AB_149924, (Lab Vision Cat# RM-9107-S1, RRID:AB_149924)
CD68
PG-M1 IgG 3, 1:200, DAKO M0876 AB_2074844, (Agilent Cat# M0876, RRID:AB_2074844)
CD20
L26 IgG 2a, 1:1000,DAKO / M0755 AB_2282030, (Agilent Cat# M0755, RRID:AB_2282030)

Clinical data

Policy information about clinical studies

All manuscripts should comply with the ICMJE guidelines for publication of clinical research and a completed CONSORT checklist must be included with all submissions.

Clinical trial registration

https://clinicaltrials.gov/study/NCT05225688?tab=history&a=1

Study protocol

https://clinicaltrials.gov/study/NCT05225688?tab=history&a=1

Data collection

We performed a prospective case-control study in the post-COVID-19 clinic of the Amsterdam University Medical Centers (UMC) and the Faculty of Behavioral and Movement Sciences (Vrije Universiteit Amsterdam). Clinical data was stored in CASTOR EDC. All patients were included in 2022.

Outcomes

https://clinicaltrials.gov/study/NCT05225688?tab=history&a=1

Primary and secondary outcomes were pre-defined based on symptomatology of post-exertional malaise. As most symptoms involve the skeletal muscles we hypothesized that skeletal muscle mitochondrial respiration was reduced while inflammation markers were elevated.

Primary outcomes were collected with:
Mitochondrial respiration data collection software: DatLab Version 7.4.0.4 (Ororoboros Instruments, Innsbruck, AT)
Metabolomics was collected using using Bruker TASQ software version 2.1.22.3.

Secondary outcomes were determined with:
Exercise tolerance: Lode Excalibur Sport, Lode, Groningen, The Netherlands
Muscle oxygenation derived via near-infrared spectroscopy (NIRS) during exercise in LONG-COVID patients: NIRS device: Portamon, Artinis Medical Systems, Arnhem, The Netherlands
